# Quality of life in older adults following a hip fracture: an empirical comparison of the ICECAP-O and the EQ-5D-3 L instruments

**DOI:** 10.1186/s12955-018-1005-9

**Published:** 2018-09-05

**Authors:** Rachel Milte, Maria Crotty, Michelle D. Miller, Craig Whitehead, Julie Ratcliffe

**Affiliations:** 1 Nutrition and Dietetics, Flinders University, Adelaide, SA Australia; 2 Institute for Choice, University of South Australia, GPO Box 2471, Adelaide SA, SA 5001 Australia; 3 Rehabilitation, Aged, and Extended Care, Flinders University, Adelaide, SA Australia; 4 Flinders Clinical Effectiveness, Flinders University, Adelaide, SA Australia

**Keywords:** Hip fractures, Quality of life, Patient reported outcome measures

## Abstract

**Background:**

The purpose of this study was to empirically compare the performance of two generic preference based quality of life instruments, EQ-5D-3 L (with a health and physical function focus) and ICECAP-O (with a wellbeing and capability focus), in a population of older Australians following hip fracture.

**Methods:**

Older adults or their family member proxies (in cases of severe cognitive impairment) following surgery to repair a fractured hip were invited to take part in this cross sectional study. Inclusion criteria included an age of 60 years or older, confirmed falls-related hip fracture and those receiving current palliative care or consented to other research studies were excluded. 87 participants completed the ICECAP-O and EQ-5D-3 L instruments between one and three weeks post-surgery.

**Results:**

For the hip fracture population, the mean ICECAP-O score was 0.639 (SD 0.206, *n* = 82) and the mean EQ-5D-3 L utility score was 0.545 (SD 0.251, *n* = 87). There was a statistically significant positive correlation between the ICECAP-O and EQ-5D-3 L scores (*r* = 0.529, *p* = < 0.001).

**Conclusions:**

Study findings indicate significant impairments in quality of life post hip fracture. In multiple regression analyses, age and health-related QoL (measured by the EQ-5D) were significant determinants of ICECAP-O scores, while proxy respondent status, age, and capability-related QoL (measured by the ICECAP-O) were significant determinants of EQ-5D scores. Approaches to measuring and valuing quality of life in this sample, should consider the target domains of the intervention in their choice of instrument. Studies aiming to measure the impact of interventions targeting broader domains of wellbeing and QoL should consider including a broader measure of QoL in conjunction with a HRQoL measure.

## Background

Long-term disability is common among patients following a hip fracture, with half of patients remaining in long-term care or needing help with basic activities of daily living one year after the fracture [1]. Thus, improvement in function, independence, and overall quality of life is a major outcome for recovery and consideration in rehabilitation strategies for older people following a hip fracture. Of the potential methods for measuring quality of life in this population, generic-preference based instruments provide many potential benefits including measurement across a number of domains of quality of life that can be applied and readily compared across different patient groups and different diseases [2, 3]. Generic preference based measures of quality of life also facilitate the calculation of utility for incorporation into economic evaluations for assessing the value for money of competing interventions.

While the concept of quality of life has been considered in part of healthcare research for some decades, much of the work to map the determinants of the concept were conducted with younger and healthy populations [4]. Potential generic preference-based measures which are widely used and demonstrate good validity and reliability include the EuroQol (EQ-5D), Assessment of Quality of Life (AQoL) and the SF-6D (which is estimated from the Short Form-36 and Short Form-12 instruments) [5]. All of these instruments measure across a range of physical health domains, and generally include aspects of mental health, social role functioning and usual activities as well. Studies of quality of life following surgery for hip fracture have been published using the EQ-5D, HUI, and SF-6D instruments [6–10]. The EQ-5D is generally found to have higher completion rates compared to the AQoL and SF-6D in older people, although the SF-6D may be more sensitive to milder health problems [11, 12].

Taking the most widely used of these instruments, the EQ-5D focuses upon five dimensions which are highly influenced by physical health and disability but which may not be sensitive to changes in other aspects of wellbeing and quality of life that are important to older people more broadly [13–15] and that are known to be reduced in addition to physical health domains following a hip fracture [16]. Therefore, investigation of instruments which measure quality of life more broadly in these social and emotional functioning domains is warranted in the hip fracture population.

The ICECAP-O (ICEpop CAPability measure for Older people) [17] is a relatively new generic-preference based instrument with a specific focus on broader aspects of quality of life and capability. Only one study to date has applied the ICECAP-O in frail older people following a hip fracture [18] and found a decline in ICECAP-O scores between pre-fracture state and four weeks following hip fracture, but no difference in scores at four months post fracture. However, participants with cognitive impairment were excluded from measurement of quality of life using the ICECAP-O, without use of a proxy. Other studies comparing the use of the EQ-5D-3 L and ICECAP-O in older adult populations have found the two instruments to be comparable in test-retest reliability, construct validity, and responsiveness in frail older adults [15, 19].

The main aim of this study was to empirically compare the relationship between two generic preference based quality of life instruments, EQ-5D-3 L (with a health and physical function focus) and ICECAP-O (with a wellbeing and capability focus), in a population of older Australians following surgery for hip fracture.

## Methods

### Quality of life instruments

The ICECAP-O [17] measures participants’ capability across five domains: attachment, security, role, enjoyment, and control, with four levels of response for each domain ranging from the worst or no capability (level 1), to the best or full capability (level 4). Valuations have been performed in a population of 255 UK older persons (over 65 years) [17] and more recently in 2456 Australian adults, both using the best-worst scaling technique [20]. Responses to the ICECAP-O can thus be converted into a single summary score, ranging from zero (indicating ‘no capability’) to one (indicating ‘full capability’).

The EQ-5D-3 L [2] covers five key dimensions; mobility, performing self-care, usual activities, pain/discomfort, and anxiety/ depression using three levels of response: from level 1 (indicating no problems or the best state for that dimension), to 3 (indicating the worst state for that dimension). Valuations have been conducted for a number of countries, [21, 22], more recently an Australian specific scoring algorithm has been created based on 414 members of the Australian general population also using a time trade off method [23], converting responses into a single summary score on a range of − 0.217 (health state worse than death) to 1 (full health). The EQ-5D also includes a sixth question, the EQ-5D Visual Analogue Scale (VAS). The EQ-5D VAS is not a preference-based measure and was not included as part of this study due to the focus on evaluating generic preference-based measures [24].

### Administration of questionnaire

The EQ-5D-3 L and the ICECAP-O were administered to participants as part of a larger cross sectional study to determine rehabilitation preferences in a group of patients following surgery to repair a fall-related hip fracture. The procedures for the study have been described previously [25]. Participants were recruited from the Flinders Medical Centre and Repatriation General Hospital in Adelaide, South Australia. The study was reviewed by the Flinders Clinical Research Ethics Committee, and approval granted February 2009 (approval number 4609). Patients were approached sequentially between May 2009 and November 2010. Inclusion criteria were an age of 60 years or older, with a radiologically confirmed falls-related hip fracture and not currently receiving palliative care or consented to another research study. In cases of significant cognitive impairment where the participant was unable to give consent or respond to the questions (defined as a score on the Mini Mental State Examination (MMSE) as less than 19 out of a possible 30) [26], consenting family members were recruited to provide their consent to the study and act as a proxy respondent.

The questions were presented to the participants during an interview with a trained research assistant, conducted between one and three weeks following surgery, to capture the initial recovery period, either at the patient bedside or at their home, with participants reporting on their (or their family member’s) health and quality of life today.

### Scoring

Individual responses to the EQ-5D-3 L and the ICECAP-O were converted into single summary scores using generic-preference based algorithms. For the EQ-5D-3 L the recently developed general population scoring algorithm was utilised based upon the EQ-5D-3 L health state preferences from Australians (*n* = 417) using a time-trade off methodology [23]. For the ICECAP-O the valuations were from an Australian general population sample (*n* = 2456) using the best-worst scaling technique [20].

### Data analysis

Responses to the EQ-5D-3 L and ICECAP-O were summarised as simple frequencies and percentages. Statistical analysis was conducted using IBM SPSS Statistics Version 19.0. Pearson’s and Spearman’s correlation coefficients were used to determine correlations between the summary scores for the two instruments and continuous and dichotomous variables, dependant on the normality of the distribution of the data. Correlations sizes below 0.3 were considered weak, those from 0.3 to less than 0.5 were considered moderate, and those from 0.5 to less than 0.6 were considered strong, and those of 0.6 or greater were considered very strong [27].Bootstrapping with 1000 iterations bias-corrected and accelerated was used to provide 95% confidence intervals for the correlation coefficients. The correlation was considered significant where the 95% confidence intervals did not cross zero.

The relationship between various demographic and clinical characteristics and the EQ-5D and ICECAP-O scores was assessed using multiple linear regression. As the purpose exploratory, to determine the influence of characteristics on the EQ-5D or ICECAP-O scores, variables were entered into the model in a single block via forced entry, including the patient gender, their age, living situation (community living = 1 vs in residential care =0), MMSE score, and proxy status (self-completed = 0 vs completed by a proxy respondent = 1), and either the EQ-5D (where the ICECAP-O score was the dependant variable) and ICECAP-O scores (where the EQ-5D was the dependant variable). All assumptions regarding multiple regression analysis were investigated using appropriate methods, including homoscedasticity, linearity, autocorrelation, normally distributed errors and multicollinearity. R^2^ are presented to indicate the proportion of variability in the dependent variable explained by the model. Standardised regression coefficients, non-standardised regression coefficients, and 95% confidence intervals of the non-standardised regression coefficients are presented. Analysis was undertaken using IBM SPSS statistics version 24.

## Results

A total of 149 patients with a recent femoral neck fracture were approached to participate, of whom 87 (58%) consented to the study. The key demographic characteristics of the participants are shown in Table 1. The vast majority of participants living in the community resided in their own home (*n* = 70, 81%). All participants completed the EQ-5D-3 L, while five participants did not complete the ICECAP-O.

**Table 1:** Demographic characteristics of the participants (*n* = 87)

Characteristics	Descriptive statistics
Number of females (%)	61 (70.1)
Mean age (SD)	80.3 (8.2)
Residential status	
Number living in HLC (%)	10 (11.5)
Number living in LLC (%)	6 (6.9)
Number living in the community (%)	71 (81.6)
Mean MMSE score (SD)	23.3 (6.9)
Number of proxy respondents (%)	10 (11.5)

*Abbreviations: HLC* High Level Care, *LLC* Low Level Care, *MMSE* Mini Mental State Examination, *SD* Standard Deviation

Table 2 gives the responses to the EQ-5D-3 L by the participants. The responses to the EQ-5D-3 L indicated that the majority of participants experienced moderate or severe impairments (response levels 2 or 3) for some or all dimensions. 79% of participants had either some problems in walking about or were confined to bed. 64% of participants also had some problems or many problems with self-care. For their usual activities, 70% of participants had either moderate or severe problems with performing their usual activities. In addition, a high percentage of participants (79%) indicated they had moderate or extreme pain or discomfort, while just under half (46%) were either moderately or extremely anxious or depressed.

**Table 2:** Distribution of responses to EQ-5D-3 L items by all participants (*n* = 87)

EQ-5D-3 L Item	Number (%)
Mobility	
I have no problems in walking about	18 (21)
I have some problems in walking about	62 (71)
I am confined to bed	7 (8)
Self-Care	
I have no problems with self-care	31 (36)
I have some problems with self-care	48 (55)
I am unable to wash or dress myself	8 (9)
Usual activities	
I have no problems with performing my usual activities	26 (30)
I have some problems with performing my usual activities	36 (41)
I am unable to perform my usual activities	25 (29)
Pain or discomfort (*n* = 85,2 participants refused to answer)	
I have no pain or discomfort	18 (21)
I have moderate pain or discomfort	62 (73)
I have extreme pain or discomfort	5 (6)
Anxiety or depression	
I am not anxious or depressed	47 (54)
I am moderately anxious or depressed	32 (37)
I am extremely anxious or depressed	8 (9)

Table 3 gives the participant responses to the ICECAP-O. The responses to the ICECAP-O also demonstrated impairments in quality of life. While 78% of the participants indicated they were able to have all or a lot of the love and friendship they wanted, and 63% that they could think about the future without any or only a little concern, the responses for the other attributes indicated the majority of participants were experiencing problems in these areas. 59% were unable to do anything or only a few things that made them feel valued. 72% stated that they could not have any or only a little of the enjoyment and pleasure they wanted. 53% were unable to be independent at all or only in a few things.

**Table 3:** Distribution of responses to ICECAP-O items by all participants (*n* = 87)

ICECAP Item	Number (%) of participants
Attachment (*n* = 86, 1 participant refused to answer)	
I cannot have any of the love and friendship that I want	2 (2)
I can have a little of the love and friendship that I want	17 (20)
I can have a lot of the love and friendship that I want	19 (22)
I can have all of the love and friendship that I want	48 (56)
Security	
I can only think about the future with a lot of concern	11 (13)
I can think about the future with some concern	21 (24)
I can think about the future with only a little concern	28 (32)
I can think about the future without any concern	27 (31)
Role (*n* = 85, 2 participants refused to answer)	
I am unable to do any of the things that make me feel valued	17 (20)
I am able to do a few of the things that make me feel valued	33 (39)
I am able to do many of the things that make me feel valued	21 (25)
I am able to do all of the things that make me feel valued	14 (17)
Enjoyment (*n* = 86, 1 participant refused to answer)	
I cannot have any of the enjoyment and pleasure that I want	10 (12)
I can have a little of the enjoyment and pleasure that I want	51 (60)
I can have a lot of the enjoyment and pleasure that I want	15 (17)
I can have all of the enjoyment and please that I want	10 (12)
Control (*n* = 84, 3 participants refused to answer)	
I am unable to be at all independent	9 (11)
I am able to be independent in few things	35 (42)
I am able to be independent in many things	30 (36)
I am able to be completely independent	10 (12)

The mean summary score value for the ICECAP-O for the sample was 0.639 (SD 0.206), and for the EQ-5D-3 L was 0.545 (0.251).

Table 4 gives the results of the correlations between the ICECAP-O and EQ-5D-3 L instrument items and their summary score values. There is evidence of a strong statistically significant correlation between the ICECAP-O and EQ-5D-3 L scores, and the EQ-5D-3 L summary score and the ICECAP-O security item. It should be noted that higher scores on the EQ-5D-3 L dimensions indicated a better state for that dimension, while a higher score on the ICECAP-O domains indicated poorer capability for that domain. There was evidence of statistically significant negative correlations between the EQ-5D-3 L mobility item and items Security, Control, and the ICECAP-O score ranging from weak to moderate in strength. For the EQ-5D-3 L self-care item there were with significant negative correlations with all ICECAP-O items except for enjoyment. There were fewer correlations seen between the EQ-5D-3 L items usual activities and anxiety and depression and the items of the ICECAP-O, and no statistically significant correlations were identified for the EQ-5D-3 L item pain and discomfort and any ICECAP-O items.

**Table 4:** Spearman correlation coefficients between ICECAP-O and EQ-5D-3 L items and summary scores

	Attachment		Security		Role		Enjoyment		Control		ICECAP-O score	
	Coefficient^a^; *p* value	95% CI	Coefficient^a^; *p* value	95% CI	Coefficient^a^; *p* value	95% CI	Coefficient^a^; *p* value	95% CI	Coefficient^a^; *p* value	95% CI	Coefficient^a^; *p* value	95% CI
Mobility	−0.218; 0.043	−0.430; 0.031	− 0.301; 0.005	− 0.473; − 0.112	− 0.246, 0.023	− 0.461; 0.006	− 0.212; 0.050	− 0.442; 0.031	− 0.277; 0.011	− 0.509; − 0.004	−0.364; 0.001	− 0.545; − 0.164
Self-care	−0.236; 0.028	− 0.439; − 0.025	−0.297; 0.005	− 0.475;-0.100	− 0.276; 0.011	− 0.497; − 0.052	−0.165; 0.130	− 0.396; 0.060	− 0.372; < 0.001	− 0.573; − 0.154	−0.387; < 0.001	− 0.593; − 0.133
Usual activities	−0.167; 0.124	− 0.377; 0.050	−0.209; 0.052	− 0.406; 0.006	−0.360; 0.001	− 0.554; − 0.188	−0.199; 0.066	− 0.408; 0.036	−0.297; 0.006	− 0.494; − 0.082	−0.362; 0.001	− 0.552;-0.145
Pain and discomfort	−0.026; 0.813	− 0.234; 0.197	−0.193; 0.078	− 0.420; 0.039	−0.025; 0.824	− 0.270; 0.232	−0.111; 0.315	− 0.335; 0.111	−0.183; 0.100	− 0.394; 0.042	−0.136; 0.229	− 0.378; 0.114
Anxiety and depression	−0.125; 0.253	− 0.334; 0.090	−0.476; < 0.001	−0.645; − 0.286	−0.165; 0.131	− 0.387; 0.078	−0.120; 0.270	− 0.366; 0.087	−0.334; 0.002	− 0.504; − 0.131	−0.368; 0.001	− 0.540;-0.168
EQ-5D-3 L score	0.267; 0.013	0.073; 0.433	0.511; < 0.001	0.315; 0.673	0.339; 0.002	0.119; 0.523	0.258; 0.016	0.028; 0.461	0.455; 0.000	0.231; 0.616	0.529; < 0.001	0.330; 0.683

^a^Spearman’s correlation coefficient, *CI* Confidence Intervals

**Fig. 1:**
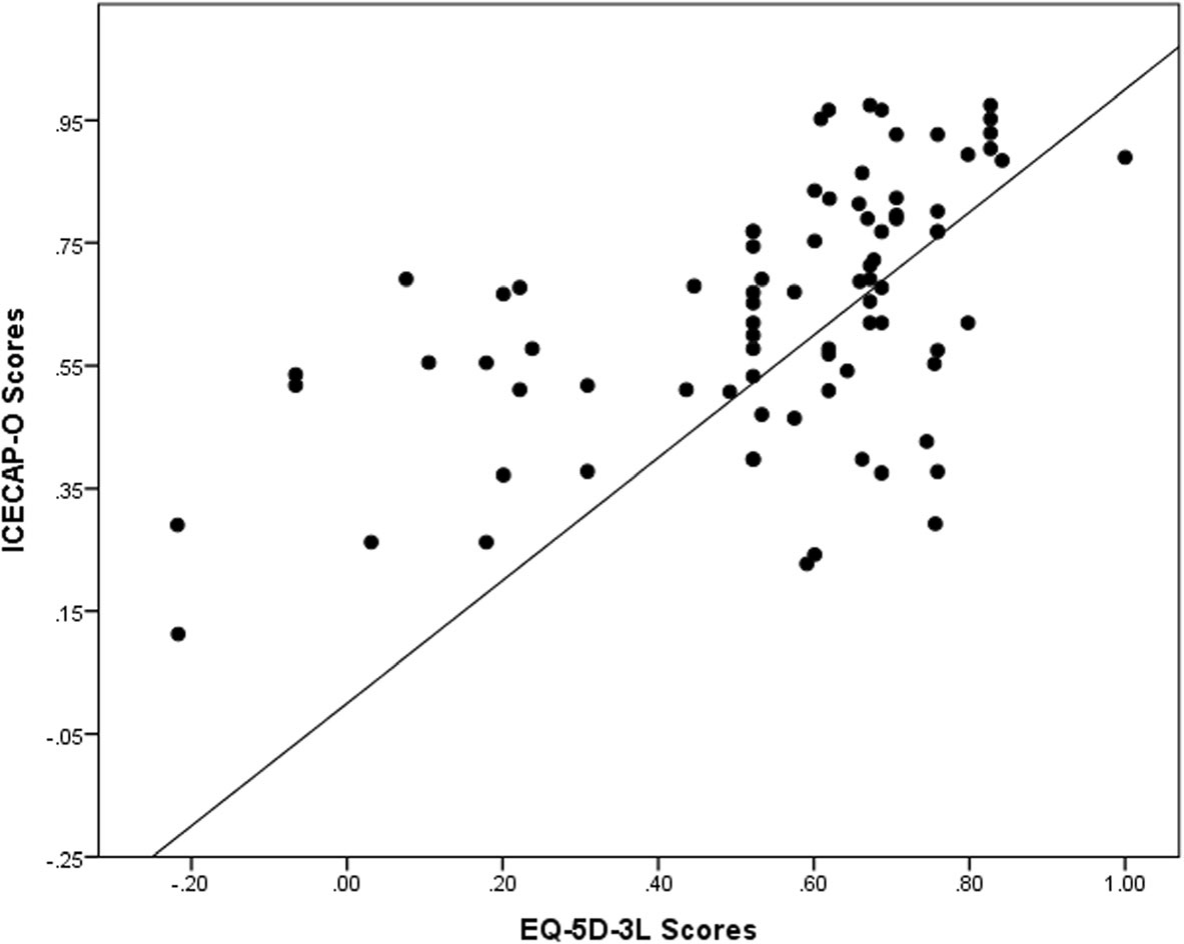
Scatterplot comparison of the ICECAP-O and EQ-5D-3 L scores. Reference line represents y = x

Figure 1 shows a scatterplot comparison of the ICECAP-O and EQ-5D-3 L, with a reference line at y = x. The points cluster around the reference line, but predominantly fall above the line indicating that scores generated using the ICECAP-O instrument are generally higher that those generated for the EQ-5D-3 L instrument.

Table 5 provides the results of the multiple regression analyses on the ICECAP-O score for the 79 participants with complete quality of life and demographic information in the study. Of the variables included in the model, the age of the participant showed a significant negative association with ICECAP-O score, while the health-related quality of life (EQ-5D) score was significantly associated with the ICECAP-O score. The other variables in the model including the gender, whether the respondent was a proxy, MMSE score for the participant, whether the patient lived in independently prior to fracture did not show significant association. Table 5 provides the results of the multiple regression analysis on the EQ-5D score. Of those variables included in the model, whether the respondent was a proxy showed a significant negative association, while the age of participants and the ICECAP-O utility both showed a significant positive association with the EQ-5D scores. The other factors included in the model including gender, MMSE score, and whether the participant resided independently prior to their fracture did not show significance.

**Table 5:** Multiple Regression analyses on ICECAP-O and EQ-5D scores

	Dependant variable: ICECAP-O scores				Dependant variable: EQ-5D-3 L scores			
Explanatory variable	B	Std. Error	95% CI of B	*p*-value	B	Std. Error	95% CI of B	*p*-value
Gender	0.059	0.043	−0.028; 0.146	0.179	0.058	0.051	−0.044; 0.160	0.260
Proxy status	0.112	0.104	−0.095; 0.319	0.282	−0.305	0.117	−0.539; − 0.071	0.011
MMSE	0.004	0.005	−0.006; 0.014	0.386	−0.008	0.006	−0.019; 0.004	0.200
Does the participant live independently?	0.035	0.058	−0.081; 0.151	0.549	0.009	0.068	−0.127; 0.145	0.897
Age	−0.005	0.002	−0.010; 0.000	0.042	0.006	0.003	0.000; 0.011	0.048
EQ5D Utility	0.414	0.088	0.240; 0.589	0.000	–	–	–	–
ICECAP-O utility	–	–	–	–	0.571	0.121	0.330; 0.812	0.000
Constant	0.576	0.257	0.063; 1.088	0.028	−0.164	0.311	−0.784; 0.457	0.601
R^2^		0.362			0.360			
Adjusted R^2^		0.270			0.307			
Number of observations	79				79			
Residual degrees of freedom	72				72			

B unstandardized regression coefficient, Description of variables: Age – continuous variable, Gender; reference level - males, Proxy status reference level, = self-completed, Does the participant live independently, reference level = No, MMSE = continuous variable, EQ-5D utility = continuous variable, ICECAP-O utility = continuous variable

## Discussion

We aimed to apply a new instrument for the measurement of quality of life (ICECAP-O) measuring broader aspects of quality of life and measure relationships with a more established health-status focused instrument (EQ-5D-3 L) in a population of older adults following a hip fracture. We identified reduced quality of life in our sample compared to values published previously for the general population and the general population of a similar age, for both instruments, and a previous study of hip fracture patients in Australia [28–31]. The majority of our sample of patients with a hip fracture indicated they had problems with the EQ-5D-3 L dimensions of mobility, self-care, usual activities, and pain or discomfort, and just under half of our sample indicated they were moderately or extremely anxious or depressed. Large proportions of our sample also indicated they had problems with the ICECAP-O attributes of role, enjoyment, and control. However, in the attributes of attachment and security, the majority of our sample indicated that they had no or only a slight impairment with these attributes.

Previous studies have investigated the determinants of QoL in hip fracture patients. A recent review identified hip fractures resulted in significantly decreased functioning and QoL following hip fracture [32]. QoL improved most significantly over the three month follow up period, with deficits at this time point like to be stable and remain at long-term follow up points. They noted that high levels of functioning prior to fracture (including good motor function, independence in living status and undertaking activities of daily living) were associated with better QoL post fracture. In contrast to our study, Buecking et al. [33] determined the factors correlated with EQ-5D score at discharge from an acute hospital among hip fracture patients. They identified significant bi-variate correlations between age, pre-fracture co-morbidity and functional status, pre-fracture residential status, MMSE on admission, type of surgery (prosthesis vs internal fixation) and presence of delirium and EQ-5D scores at discharge. In the multiple-regression analysis only significant relationships with residential care prior to fracture, MMSE scores, and type of surgery remained.

This study represents a new application of the ICECAP-O instrument measuring broad aspects of quality of life in a sample of older people following a hip fracture for the first time previous studies having focused on community dwelling, transition care, or residential aged care populations [15, 28–30] [19, 34–37]. One previous study has reported ICECAP-O in a population following hip fracture with a mean score of 0.73 (SD 0.22) at four weeks following fracture among 59 people excluding those with cognitive impairment, higher than the value reported in our study. By comparison, cur current sample included responses from participants with cognitive impairment themselves where possible or included a response from a proxy on behalf of residents with more severe cognitive impairment.

Our study also represents a unique contribution in the comparison of the ICECAP-O with more established health-state focused measures of quality of life such as the EQ-5D-3 L. The results of the correlation show small to moderate correlations between the newly developed ICECAP-O and the more established EQ-5D-3 L however the ICECAP-O summary score was found to be generally higher than that generated from the EQ-5D-3 L. In addition, there was evidence of weak or negligible correlations between some items of the instrument, particularly the pain and discomfort and anxiety and depression items of the EQ-5D-3 L, and the attachment, role and enjoyment items of the ICECAP-O, indicating disparity in the domains measures between the two instruments. Hypothetically we expected that experiencing pain and discomfort would reduce your ability to carry out your role, or have enjoyment in life, and that people with higher levels of attachment would report lower levels of anxiety and depression. In our study, correlations between the measures were in these expected directions, however the associations were weak or negligible. Similar results have been found in other studies comparing the two instruments [15, 18, 19, 35, 38].

Further work is needed to understand the relationship between different domains of quality of life and how they are impacted and recover overtime post hip fracture. Previous studies conducted in older people attending a falls clinic have indicated the ICECAP-O and EQ-5D-3 L capture distinct domains of quality of life, and thus are complementary [38]. In addition, a study applying both instruments in the generation of QALYs for economic evaluation of an integrated care model for frail elderly found that the intervention was more likely to be considered cost effective with use of the ICECAP-O than the EQ-5D-3 L, indicating the instrument may be more suitable for assessing impact of interventions where the focus is on social and psychological wellbeing [39]. Others have called for the use of multiple instruments in evaluations of interventions for older people, measuring both health-related quality of life as well as broader aspects of quality of life and wellbeing [15, 16].

There are a number of limitations to the study design. As the study was cross-sectional in nature, we are unable to determine the sensitivity of these measures to changes in health status and wellbeing overtime, which is important in choosing an instrument to measure the impact of novel healthcare interventions in this group. The study also recruited only a small proportion of the total population of patients admitted with hip fractures, with a low recruitment rate (58%) of those eligible for the study, limiting the generalisability of these findings to the wider population of patients with a hip fracture. Admission with a hip fracture is a time of great stress for patients and their families which may have impacted on the recruitment rate for this study. Future studies offering rewards or conducted as part of intervention studies may see improved recruitment rates. While we attempted to interview participants once their clinical status had stabilised post-surgery, in practice these interviews occurred between 1 and 3 weeks post fracture. Interviews were conducted at a time that suited patients or family members during this stressful time to try and increase likelihood of participation. However, it should be noted that at this time patient’s recovery can be variable – with some recovering function quickly while others remain in poor function. Future studies which are more selective of the timeframe patients are admitted into the study would be useful to provide a clearer picture of the level of recovery in QoL at this time.

In this study due to significant cognitive impairment we were not able to elicit responses from those individuals with MMSE scores lower than nineteen. However, there is currently no universally agreed threshold level of cognitive impairment beyond which proxy responses should be sought [40]. Available evidence shows proxy responders tend to overestimate limitations in unobservable items (e.g. pain, anxiety) for measuring health-related quality of life making simple substitution problematic [40]. The involvement of people with cognitive impairment and proxies in reporting quality of life is deserving of continuing research focus.

## Conclusions

This paper has provided exploratory data of the use of a new generic preference based instrument for measuring quality of life (ICECAP-O) specifically designed for use in older people. The ICECAP-O demonstrated good feasibility in this population. We identified that quality of life as measured in a broader sense by the ICECAP-O demonstrated a positive association with health-related quality of life (as measured by the EQ-5D-3 L) in a population of older people post hip fracture. However there is evidence of differences in agreement between the two instruments, which highlights the potential difference in recovery of health status and broader quality of life in this population. Future studies should evaluate the change overtime in broader quality of life, as measured by instruments such as the ICECAP-O, in patients following a hip fracture to inform a full understanding of recovery in this population.

## Abbreviations

EQ-5D-3 L: EQ-5D Three Level version
HLC: High Level Care
ICECAP-O: ICEpop CAPability measure for Older people
LLC: Low Level Care
MMSE: Mini Mental State Examination
SD: Standard Deviation
UK: United Kingdom
